# A combinatorial screening protocol for identifying novel and highly potent dual-target inhibitor of BRD4 and STAT3 for kidney cancer therapy

**DOI:** 10.3389/fphar.2025.1560559

**Published:** 2025-02-26

**Authors:** Shizhu Zhang, Nan Wu, Yifei Geng, Lixia Guan, Miao-Miao Niu, Jindong Li, Lusha Zhu

**Affiliations:** ^1^ Department of Nephrology, Huai’an Cancer Hospital, Huai’an, China; ^2^ Department of Pharmaceutical Analysis, China Pharmaceutical University, Nanjing, China; ^3^ Department of Pharmacy, Taizhou School of Clinical Medicine, The Affiliated Taizhou People’s Hospital of Nanjing Medical University, Taizhou, China

**Keywords:** renal cell carcinoma, bromodomain-containing protein 4 (BRD4), signal transductor and activator of transcription 3 (STAT3), virtual screening, dual-targeted inhibitors

## Abstract

Concurrent inhibition of bromodomain-containing protein 4 (*BRD4) and* signal transductor and activator of transcription 3 (STAT3) could potentially be an effective strategy against renal cell carcinoma (RCC). Here, we successfully identified five dual-targeted BRD4/STAT3 inhibitors (BSTs 1–5) using a combinatorial screening protocol. Particularly, BST-4 was the most potent inhibitor simultaneously targeting BRD4 (IC_50_ = 2.45 ± 0.11 nM) and STAT3 (IC_50_ = 8.07 ± 0.51 nM). MD simulation indicated that BST-4 stably bound to the active sites of BRD4 and STAT3. The cytotoxicity assays exhibited that BST-4 had a significant antiproliferative activity against RCC cell lines, especially CAKI-2 cells (IC_50_ = 0.76 ± 0.05 μM). Moreover, *in vivo* experiments revealed that BST-4 more effectively inhibited the growth of xenograft tumors compared with positive controls RVX-208 and CJ-1383. Overall, these data indicated that BST-4 could be a promising candidate compound for RCC therapy.

## 1 Introduction

Kidney cancer accounts for 3.8% of all cancer burden worldwide and is the seventh most common cancer in men and the ninth most common cancer in women (Gupta et al., 2011; Greef and Eisen, 2016; Rossi et al., 2018). More than 400,000 new patients are diagnosed with kidney cancer worldwide each year (Yong et al., 2019). Renal cell carcinoma (RCC) is the most common solid tumor of the kidney and the third most common malignant tumor of the urinary system (Shuch et al., 2015). However, the lack of effective drugs for treating kidney cancer so as to improve the survival of patients prognosis (Rini et al., 2019). Traditional therapies, such as surgery, chemotherapy, and radiotherapy, have their limitations (Kaur et al., 2023). Surgical resection is usually the first-line treatment for early-stage RCC, but a significant number of patients have advanced or metastatic disease at the time of diagnosis, making surgery less effective (Bhatt and Finelli, 2014). In addition, chemotherapy and radiotherapy have limited efficacy in RCC due to the fact that renal cancer cells are intrinsically resistant to many chemotherapeutic agents and relatively resistant to radiation (Corrò and Moch, 2018). Thus, the current treatment landscape for RCC is fraught with difficulties. Targeted therapies have emerged as an important approach in recent years. Single target therapies are often unable to completely block the complex signaling pathways involved in RCC progression (Jin et al., 2023). Tumor cells can easily adapt by activating alternative pathways. In this context, dual-targeting therapy offers a glimmer of hope (Taghipour et al., 2022). By simultaneously targeting two key signaling pathways involved in RCC development and progression, the development of novel dual-targeting drugs may change the landscape of the RCC therapy field.

**FIGURE 1:**
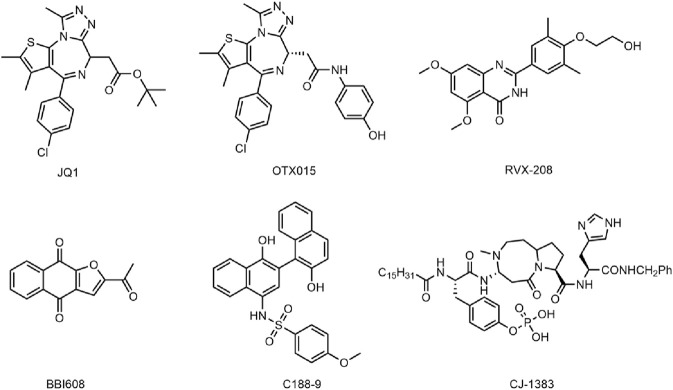
Reported BRD4 and STAT3 inhibitors.

Bromodomain-containing protein 4 (BRD4), a member of the BET family, affects cell cycle progression by activating oncogenes such as c-MYC, JUNB, CCND1 and CCNA1, thereby selectively interfering with mediating cancer cell growth and escaping apoptosis (Dawson et al., 2011; Baratta et al., 2015). BRD4 proteins play an important role in coordinating normal development and maintaining oncogene expression (Dawson, 2017). It has been shown that BRD4 proteins are involved in the development of a variety of cancers, and their alterations are considered to be an important oncogenic driver that causes or maintains malignant growth, so BRD4 has become an attractive cancer therapeutic target (Fong et al., 2015; Bechter and Schoffski, 2020; He et al., 2020; Andrikopoulou et al., 2021). At present, many BRD4 small molecule inhibitors have been developed with good antitumor activity, including JQ1 (Wang et al., 2020), OTX015 (Berthon et al., 2016), RVX-208 (McLure et al., 2013) (Figure 1) and so on. JQ1 has been shown to exert anticancer effects by inhibiting cell proliferation and inducing myeloid differentiation. However, JQ1 has the disadvantages of poor pharmacokinetic profile and low oral bioavailability (Zuber et al., 2011). OTX015 and RVX-208 have been shown to be effective in a variety of tumors (Chaidos et al., 2014; Boi et al., 2015; Xie et al., 2018), but these patients have shown serious side effects, including gastrointestinal disorders, thrombocytopenia, hyperbilirubinemia, fatigue, headache (Lewin et al., 2018; Piha-Paul et al., 2020). In addition, another major problem with BRD4 inhibitors is that drug resistance to BRD4 inhibitors often appears in various cancer types with different mechanisms of action, especially as BRD4 inhibitors are less effective in solid tumors than in hematological malignancies (Jin et al., 2018; Pawar et al., 2018; Wang W. et al., 2021). Studies have shown that SPOP mutations lead to impaired and upregulated degradation of BRD4 protein, thus conferring intrinsic resistance to BRD4 inhibitors (Dai et al., 2017; Janouskova et al., 2017). Increased Wnt/β-catenin signaling leads to reactivation of MYC expression and ultimately resistance to BRD4 (Fong et al., 2015; Guo et al., 2021). Therefore, based on the severe limitations of BRD4 monotherapy in clinical application, the use of dual-targeting and combining drug delivery regimens to improve the efficacy of BRD4 inhibitors have attracted increasing attention (Pham et al., 2019; Yan et al., 2019; Principe et al., 2022).

The signal transductor and activator of transcription (STAT) protein is a multifunctional transcription factor involved in a variety of biological processes (Darnell et al., 1994; Zou et al., 2020). The signal transducer and activator of transcription 3 (STAT3) plays a pivotal role in cell proliferation, angiogenesis, and immunosuppression, and its overactivation often leads to the development of cancer and poor clinical prognosis (Johnson et al., 2018; Hanlon et al., 2019; Ouyang et al., 2022). STAT3 is constitutively activated in cancer cells and tumor microenvironment cells due to overactivation of cytokine and growth factor pathways, which is caused by receptor and non-receptor tyrosine kinase mutations and the defects of SOCS protein (Wingelhofer et al., 2018; Liu et al., 2023). The activated STAT3 can upregulate mRNA levels of many genes involved in cell growth and apoptosis, such as cyclin D1, D2, D3, which cooperate to induce carcinogenic transformation of cells (Tolomeo and Cascio, 2021). In addition, STAT3 has been widely identified by numerous studies as a carcinogenic transcription factor that causes malignant transformation, so STAT3 has been considered an attractive target for the treatment of cancer (Zhou et al., 2019; Ma et al., 2020; Mohan et al., 2022). The reported inhibitors of STAT3 such as BBI608 (Napabucasin) (Zhang et al., 2016), C188-9 (Silva et al., 2015; Bharadwaj et al., 2016) and CJ-1383 (Chen et al., 2010) (Figure 1), directly target STAT3 by disrupting the domain of SH3, DBD, or NTD (Beebe et al., 2018). However, most inhibitors have poor clinical efficacy due to low water solubility and low cell permeability (Beebe et al., 2018). Therefore, it is necessary to develop novel and effective STAT3 inhibitors to avoid the above problems.

BRD4 inhibitors and STAT3 inhibitors as monotherapies have limitations in terms of therapeutic activity, drug resistance and side effects (Beebe et al., 2018; Tian et al., 2019; Zhang et al., 2019). Acquired resistance to BETis is poorly understood in the clinical setting, but it has been investigated in recent preclinical studies that resistance to BET inhibitors targeting BRD4 is common in solid tumors (Cochran et al., 2019). Dual-target therapy may expand the therapeutic index, and display either synergistic effects or synthetic lethality that may reduce the drug resistance frequently seen with monotherapy during cancer treatment (Cochran et al., 2019; Tang et al., 2021). Recent studies have found that BRD4 provides chemoresistance by inhibiting apoptosis through STAT3 activation prior to enhanced DNA damage repair (Pecharromán et al., 2023). BRD4 protein phosphorylation promotes interaction with STAT3, and confers increased oncogenic enhancer activity, necessitating co-targeting strategies to eliminate or reverse this process. The development of dual-targeting BRD4/STAT3 inhibitors may improve anticancer efficacy and provide new insights to address drug resistance (Ray et al., 2014). In addition, dual-targeted drugs can more precisely target tumor cells, theoretically enhancing the tumor selectivity of the drug (Schubert et al., 2012). Moreover, dual-targeted therapies have lower targeting toxicity and side effects compared with combination therapies (Taghipour et al., 2022). Therefore, we aim to develop a novel and effective dual-targeting BRD4/STAT3 inhibitor to avoid the liability arising from monotherapy and combination therapy and provide a new option for cancer treatment. Furthermore, to our knowledge, no inhibitors targeting both BRD4 and STAT3 have been reported.

Virtual screening has become a valuable computer technology that can greatly improve screening efficiency and reduce expenses compared to traditional high-throughput screening of drugs (Schröder et al., 2013; Chen et al., 2014). Our previous studies have used virtual screening to successfully identify a dual-targeting tubulin/PARP-1 inhibitor and a cyclic peptide targeting NRP1 and KRAS^G12D^ for cancer therapy (Wang S. et al., 2021; Zhou et al., 2022). Here, we aimed to discover a novel potent dual-targeting BRD4/STAT3 inhibitor through a combinatorial screening protocol. First, a pharmacophore model was constructed based on BRD4 and STAT3 crystal structures. Potential compounds were retrieved from database through the pharmacophore model. The compounds were then docked to BRD4 and STAT3, and five compounds (BSTs 1-5) were selected according to docking scores that were better than controls. Finally, the activity of *in vitro* assay and *in vivo* xenograft tumor model evaluation of hit compounds was further evaluated. Among them, BST-4 is the most potential compound with the strong inhibitory effect and low toxicity which may become a promising compound for RCC therapy in the future.

## 2 Materials and methods

### 2.1 Reagents

All compounds were acquired from WuXi AppTec (Shanghai, China), solubilized in DMSO and added at the indicated concentrations. Phosphate-buffered saline (PBS), Dulbecco’s modified Eagle’s medium (DMEM), Roswell Park Memorial Institute Medium (RPMI) 1,640, McCoy’s 5A medium, fetal bovine serum (FBS) and penicillin/streptomycin were purchased from Gibco (Grand Island, New Nork, USA). BRD4 and STAT3 proteins were obtained from Abcam (Cambridge, MA, United States).

### 2.2 Cell lines and culture condition

Human normal renal cortical proximal tubular epithelial cell line HK-2 and human renal carcinoma cell lines CAKI-2, SLR-23, 769-P, A704 and RCC4 were previously purchased from the American Type Culture Collection (ATCC, Manassas, VA, USA) or the European Collection of Cell Cultures (ECACC, Salisbury, United Kingdom). HK2, A704, and RCC4 cells were maintained in high glucose DMEM supplemented with 10% FBS and 1% penicillin/streptomycin. SLR-23 and 709-P cells were cultured in RPMI-1640 medium with 10% FBS and 1% penicillin/streptomycin. CAKI-2 cells were maintained in McCoy’s 5A medium supplemented with 10% FBS and 1% penicillin/streptomycin. All the cell lines were cultured at 37°C in a humidified atmosphere of 5% CO_2_ and 95% air.

### 2.3 Constructing pharmacophore models

First, the crystal structure of the BRD4 (PDB ID: 5UVX) with ligand was obtained from the Protein Data Bank (PDB). PDB file was imported into the Molecular Operating Environment (MOE, Chemical Computing Group Inc, Montreal, Quebec, Canada) software. Subsequently, we pre-processed the crystal structure through the QuickPrep tool using Amber10: EHT force field, including energy minimization, addition of hydrogen atoms, and supplementation of missing residues. The pharmacophore model was constructed using Pharmacophore Query Editor of MOE. When selecting pharmacophore features, key intermolecular interactions of the target protein and the ligand were analyzed by the Ligand Interactions module of MOE. The criteria for the construction of the pharmacophore feature were to use the structure of the original ligand of BRD4 (A-1359643) (PDB ID: 5UVX) as a binding and positional reference to establish a motif characterization of the ligand binding to the BRD4 residue at the key binding site of the ligand. Hydrogen bonding features are determined on the basis of geometric and energetic criteria, in which a hydrogen bond donor feature is established on a partially positively charged hydrogen atom forming a hydrogen bond with a protein residue, and vice versa for a hydrogen bond acceptor feature. Aromatic features are established on benzene ring groups forming aromatic-aromatic interactions with protein residues. The final generated pharmacophore models were used for subsequent virtual screening.

### 2.4 Virtual screening

The crystal structures of BRD4 (PDB ID: 5UVX) and STAT3 (PDB ID: 6NUQ) proteins in complex with ligands were obtained from the Protein Data Bank (PDB) and imported into the Molecular Operating Environment (MOE). Protein preparation was performed using QuickPrep tool in Amber 10: EHT forcefield, including energy minimization, addition of hydrogen atoms, and supplementation of missing residues. An in-house database of 43,000 compounds was imported into MOE, 3D conformations of compounds generated through Conformational Search, and energy optimization of structures using MMFF94x forcefield through Energy Minimization. Pharmacophore-based virtual screening based on the pharmacophore model established above. Next, these compounds screened were further docked in MOE. By applying the Dock tool of MOE, each compound was docked to BRD4 and STAT3. The docking was determined to use the Triangle Matcher method and the London dG scoring algorithm. The docking score was used to evaluate the affinity of compounds binding to target proteins.

### 2.5 AlphaScreen assays

The inhibitory effect was measured through the previously described the amplified luminescent proximity homogeneous assay (AlphaScreen) (Feng et al., 2018). First, the compounds were diluted twofold to different concentrations (64, 32, 16, 8, 4, 2, 1, 0.5, 0.25, 0.125, 0.0625 nM) and each was incubated with BRD4 protein at a concentration of 12.5 nM for 15 min at room temperature. Biotin-labeled peptide (SGRG-K(Ac)-GG-K(Ac)-GLG-K(Ac)-GGA-K (Ac)-RHRKVGG-K-biotin) was added for 10 min, followed by streptavidin donor beads and AlphaScreen nickel chelate acceptor and incubation for 1h. All components were incubated in a total volume of 20 μL assay buffer containing 20 mM HEPES, pH 7.4, 0.1% bovine serum albumin (BSA, w/v), 0.01% Triton X-100, and 1 mM DTT. Finally, counts were measured by EnVision (Perkin Elmer, Hopkinton, MA) and analyzed by Graphpad Prism 7.0 software.

### 2.6 MD simulation

The BRD4-BST-4 and STAT3-BST-4 complexes were simulated using GROMACS 2021.5 in the AMBER99SB-ILDN force field with periodic boundary conditions. Firstly, in a cubic box located 1.0 nm away from the complex, the complex is dissolved with a SPC water molecules, and the system is neutralized by replacing water molecules with Na^+^ and Cl^−^. Using the steepest descent algorithm to minimize energy in the system for 1,500 steps. The NVT simulation was further carried out with a 100 ps V-rescale thermostat to m keep the temperature at 300 K. A Parinello-Rahman barostat was used to conduct a 100 ps NPT simulation while keeping the system pressure at 1 bar. Finally, 50 ns MD simulations were carried out on the system. Every 10 ps, trajectory data were stored.

### 2.7 Microscale thermophoresis (MST) assays

The MST experiments were performed using the Monolith NT.115 instrument. BRD4 and STAT3 proteins were labeled with the Lys labeling kit at the final concentration of 100 nM. The compounds, previously suspended in 100% DMSO, were diluted in a buffer containing 20 mM Tris·HCl (pH 7.5), 100 mM NaCl, and 3 mM DTT, achieving a final DMSO concentration of 5%. The compounds were gradient-diluted in 1:1 dilution and starting from 100 μM. The samples were loaded into MST-standard glass capillaries. All the measurements were performed in triplicate using automatically assigned 50% MST power and 20% LED power. Data analysis was performed using GraphPad Prism 7.0 software.

### 2.8 NanoBRET assays

The method was performed as previously reported (Horai et al., 2024). Briefly, HEK293T cells were seeded in dishes and allow the cells to attach and recover for overnight. The mixture of Histone H4 HaloTag Fusion vector DNA, NanoLuc BRD4/STAT3 FL Fusion Vector DNA, opti-MEM, and FuGENE HD was added dropwise to the HEK293T cells followed by incubation for 20–24 h at 37°C and 5% CO_2_ and allow the proteins to express. The cells were collected and re-seeded in 384 well plates. HaloTag NanoBRET 618 Ligand was added to plate and allow the cells to attach and recover for 4 h. Serially diluted compounds were transferred to the plate and incubated the plate overnight. Then NanoBRET Nano-Glo Substrate in Opti-MEM was added, and shake plate to mix. The donor emission (450 nm) and acceptor emission (610 nm), BRET signal, were measured within 10 min of substrate addition using microplate reader (Envision, PerkinElmer). Data analysis was done by measuring the ratio of acceptor emission to donor emission (BRET ratio). IC_50_ values and curve fits were obtained using GraphPad Prism 7.0 software.

### 2.9 Kinase selectivity profile

The kinase selectivity profile of BST-4 was performed by the SelectScreen kinase profiling service (Thermo Fisher Scientific). Kinase selectivity assay was performed using Z′-LYTE ™ screening protocol kit. Briefly, the test compounds were screened in 1% DMSO (final). For 10-point titrations, perform a 3-fold dilution starting at a starting concentration of 50 µM. Then 2.5 µL 4× test compound, 2.4 µL kinase buffer, 5 µL 2× kinase mixture and 2.5 µL 4× ATP solution was added in black 384-well plate. After shaking for 30 s, the plates were incubated for 60 min at room temperature. Add developing reagent solution and shake the plate for 30 s, then incubate for 1 h at room temperature. The Fluorescence intensity per well was read using a fluorescence plate reader. SelectScreen^®^ Kinase Profiling Service used XLfit from IDBS. The dose response curve was curve fit to model number 205 (sigmoidal dose-response model) and inhibition rates were calculated. 50% inhibition was used as a activity threshold. IC_50_ values were calculated based on curve fitting.

### 2.10 Western blot assay

For the assessment of protein expression levels, Western blot analysis was conducted following a standardized protocol (Zhou et al., 2022). The CAKI-2 cells were initially rinsed with PBS to remove any residual culture medium. Subsequently, cells were lysed using a RIPA buffer supplemented with 1 mM phenylmethylsulfonyl fluoride (PMSF), and the lysate was incubated on ice for a duration of 1 h. The lysate was centrifuged at a speed of 13,000 rpm for 30 min at 4°C. Protein concentrations were quantified using a BCA protein assay kit (Beyotime, Shanghai, China), ensuring equal protein loading for each sample. Samples were then resolved by SDS-PAGE and transferred onto a polyvinylidene difluoride (PVDF) membrane. Subsequently, the membrane was blocked with 3% BSA in PBS at room temperature for 2 h and incubated overnight with the corresponding antibody at 4°C. After washing to remove unbound primary antibodies, the membrane was incubated with horseradish peroxidase (HRP)-conjugated secondary antibodies for a period of 1 h at room temperature. The specific protein bands were visualized using ECL detection system (Tanon, Shanghai, China), and the band intensities were quantified using ImageJ software.

### 2.11 *In vitro* cytotoxicity assay

The effect of hit compounds on cytotoxicity was determined as previously reported (Ding et al., 2020). In brief, cells treated with the tested compounds were added with MTT reagent to form formazan crystals, and then the optical density (OD) values at 570 nm were determined after the crystals were dissolved in DMSO. Here, in order to initially screen the most potent candidate compounds, the inhibition rates of all compounds on RCC Cell lines from the European Collection of Cell Cultures (ECACC, Salisbury, United Kingdom) were determined at the same concentration. Next, the values of IC_50_ of the selected compounds against more RCC cell lines were determined. The 5 × 10^3^ cells in 100 μL culture medium were injected into each well of a 96-well plate and cultured overnight. Add different concentrations of compounds and incubate for 48 h. Then 100 μL of MTT (0.5 mg/mL) solution was added to the 96-well plate and maintained at 37°C for 4 h. Finally, 200 μL of DMSO was added and shaken for 10 min. The OD values at 570 nm were determined by a Synergy 4 microplate reader.

### 2.12 Quantitative real Time-PCR (RT-qPCR) analyze

This method was performed as previously reported. Briefly, the CAKI-2 cells were seeded into 6-well plates and cultured overnight, and treated with different concentrations of BST-4 for 24 h. RNA was extracted from cells and tissues using Trizol reagent and reverse transcribed using PrimeScriptRTase. cDNA was used for RT-qPCR SYBR Green assays (Takara). The relative levels of c-myc mRNA were calculated as [(c-myc mRNA in the treatment group)/(β-actin mRNA in the treatment group)]/[(c-myc mRNA in the control group)/(β-actin mRNA in the control group).

### 2.13 *In vivo* antitumor assay

To further investigate the *in vivo* effects of BST-4, 6–8 weeks male BALB/c nude mice were obtained by Changzhou Cavens Experimental Animal Co., Ltd. (Changzhou, China). The mice were acclimatized to the laboratory environment for a week before the experiment. The housing conditions were maintained at a temperature of 22°C ± 2°C, a relative humidity of 50%–60%, and a 12-h light/dark cycle. 0.1 mL of CAKI-2cell suspension (1 × 10⁶ cells) was subcutaneously injected into the right flank of each mouse. When the tumor size reached 90–120 mm^3^, mice were randomly divided into four groups (5 mice in each group): vehicle group, BST-4 group (10 mg/kg), RVX-208 group (10 mg/kg), and CJ-1383 group (10 mg/kg), and injected intraperitoneally every 12 h. The vehicle group was injected with a vehicle solution consisting of 5% DMSO and PBS. The tumor volume of mice was measured every 2 days and calculated according to the formula: (c × c × d)/2, where c and d represent the minimum diameter and maximum diameter, respectively. All animal experiments were performed and approved by the Ethics Committee of China Pharmaceutical University.

### 2.14 Statistical methods

A two-tailed Student’s t-test was used to test statistical significance. The p-values were calculated with GraphPad Prism 7.0 software. IC_50_ values were calculated with GraphPad Prism 7.0 software using a nonlinear regression model. All data were obtained from three independent experiments performed in triplicate, and the results are presented as mean and standard error of the mean.

## 3 Results and discussion

### 3.1 Virtual screening

**FIGURE 2:**
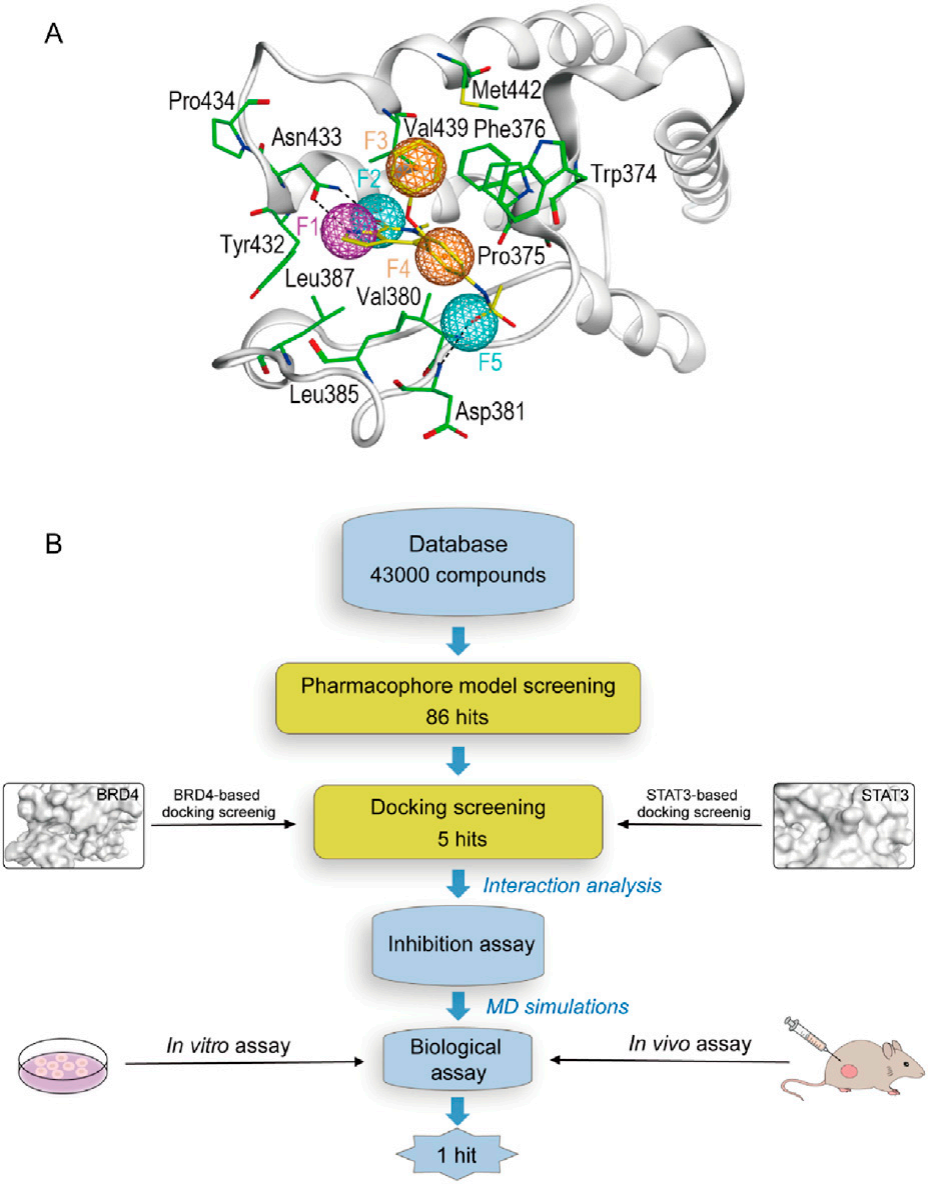
**(A)** The pharmacophore model based on the BRD4 structure. **(B)** The workflow of multi-step virtual screening and biological evaluation of dual BRD4/STAT3 inhibitors.

In order to obtain all available chemical and structural information on the binding modes of the ligands to BRD4 and STAT3, respectively, pharmacophore models were established according to the structure of BRD4 using the MOE software. Firstly, the interaction between protein and ligand was analyzed by the Ligand Interactions tool. The ligand group forms hydrogen bonding interactions with Asn433 and Asp381. The benzene ring capable of forming considerable hydrophobic interactions with Pro375, Trp374, Phe376, Leu385, Leu387, Val439 and Val380 residues. As presented in Figure 2A, four pharmacophore features were established: a hydrogen-bond donor feature (F1: Don), two hydrogen-bond acceptor features (F2 and F5: Acc) and two aromatic features (F3 and F4: Aro). These features correspond directly to some of the key amino acids of the binding site, which play a critical role in BRD4 inhibitory activity.

**FIGURE 3:**
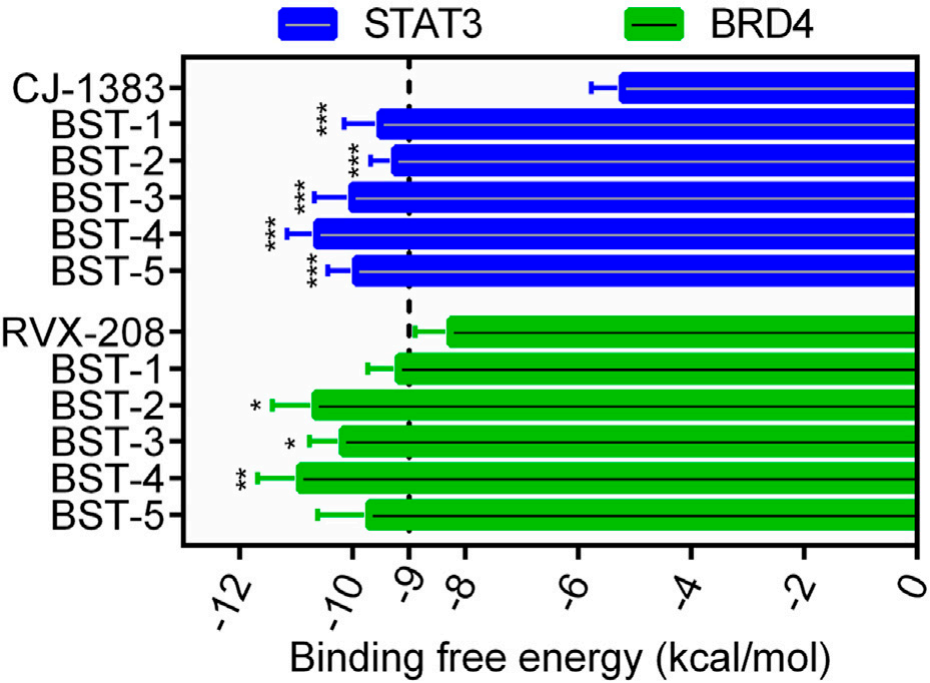
The binding free energy (kcal/mol) of five selected hit compounds (BSTs 1-5). (The *P* value was determined by a 2-tailed paired t-test: STAT3, ***p < 0.001 vs. CJ-1383; BRD4, *p < 0.05 vs. RVX-208, and **p < 0.01 vs. RVX-208).

**FIGURE 4:**
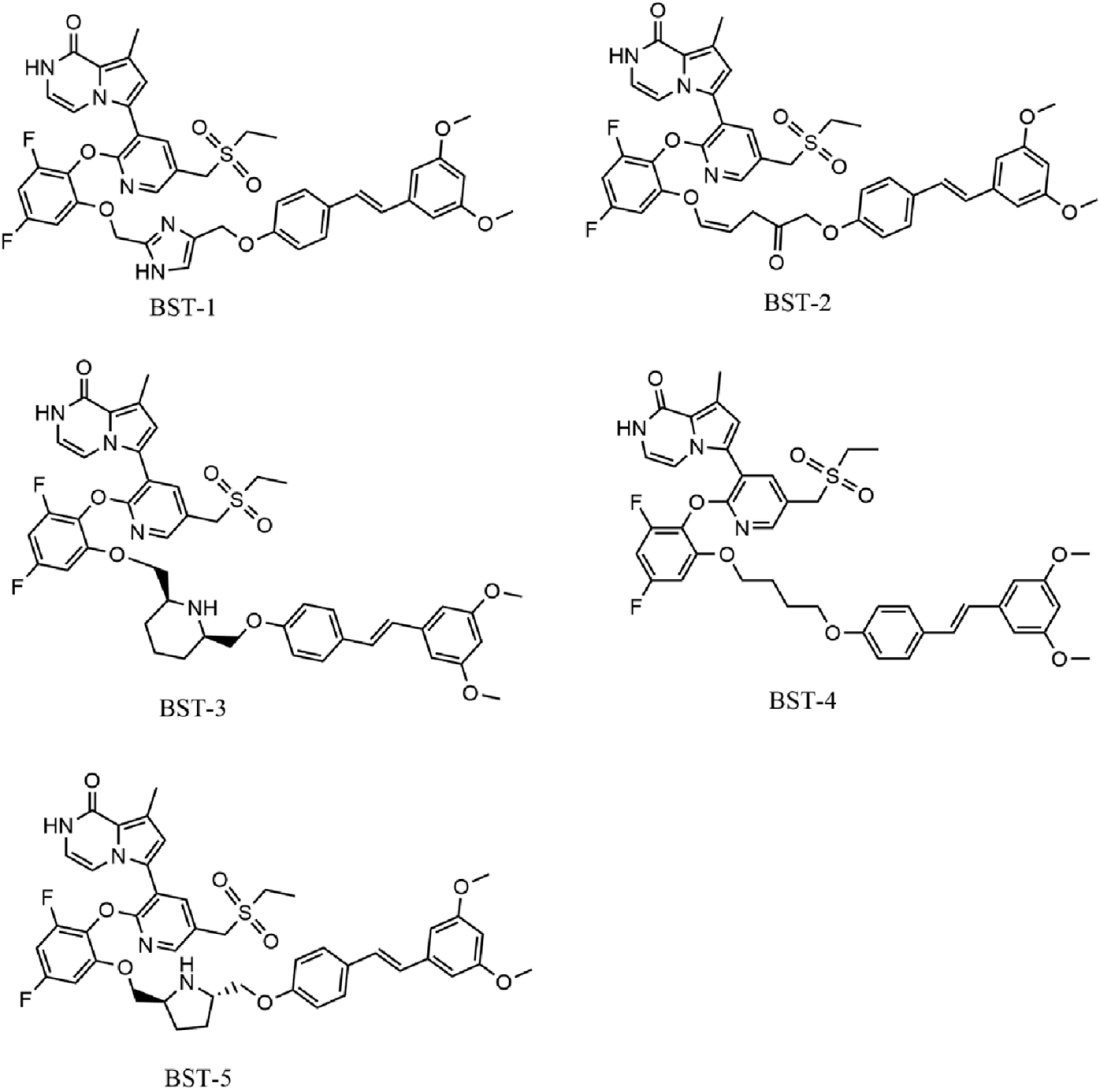
The chemical structures of five selected hit compounds (BSTs 1-5).

Figure 2B depicted the process of the virtual screening process in this investigation to identify novel dual BRD4 and STAT3 targeted inhibitors from a constructed compound database. The pharmacophore model created above was used to screen potential inhibitors from a database containing 43,000 compounds. The 86 hits obtained after virtual screening based on pharmacodynamics were subsequently screened by BRD4 and STAT3-based molecular docking respectively. Docking scores were used to evaluate the binding affinity of compounds for both BRD4 and STAT3, where lower value denoted higher binding affinity. As illustrated in Figure 3, the compounds were examined according to the binding free energies. For the docking results of BRD4, we chose −9 kcal/mol as the cutoff value and selected a set of top-ranked compounds with binding free energies below −9 kcal/mol. For STAT3 docking results, the top-ranked compounds were identified using a docking score threshold of −9 kcal/mol. Ultimately, the top five hits (termed as BSTs 1-5) that simultaneously satisfied the above docking cutoff values were screened for further affinity testing. The structures of the BSTs 1-5 were displayed in Figure 4.

### 3.2 Inhibitory effects BSTs 1-5 on BRD4 and STAT3

To further investigate the binding ability of BSTs 1-5 to BRD4 and STAT3, we performed BRD4 and STAT3 binding inhibition assays. Two other known BRD4 inhibitor (RVX-208) and STAT3 inhibitor (CJ-1383) served as positive controls. As shown in Table 1; Supplementary Figure S1, all compounds showed nanomolar levels of inhibitory activity, and their IC_50_ values were all less than those of the BRD4 and STAT3 positive control inhibitors, respectively. Among BSTs 1-5, BST-4 showed the strongest inhibitory effect on both BRD4 (IC_50_ = 2.45 ± 0.11 nM) and STAT3 (IC_50_ = 8.07 ± 0.51 nM). In particular, it inhibited BRD4 about 16-fold more than that of RVX-208, and STAT3 about 115-fold more than that of CJ-1383. Subsequently, we not only further evaluated the biological activity of BST-4, but also analyzed the stability of its targeted binding by MD simulations.

**TABLE 1:** Structure−activity relationships (SARs) of BSTs 1–5.

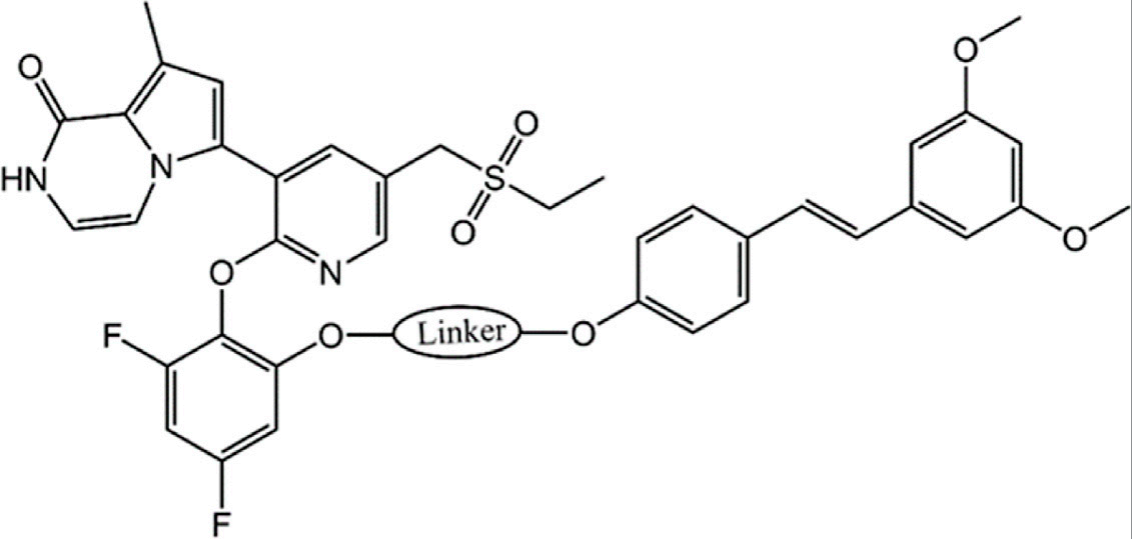			
Compounds	Linker	BRD4 (IC_50_, nM)	STAT3 (IC_50_, nM)
BST-1	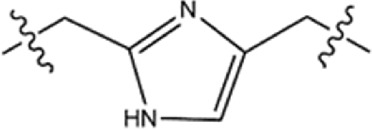	15.73 ± 1.31	20.82 ± 1.94
BST-2	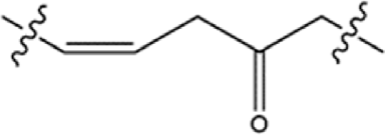	4.64 ± 0.17	27.16 ± 2.82
BST-3	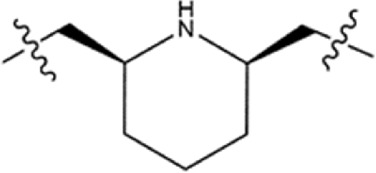	7.09 ± 0.53	11.33 ± 0.95
BST-4	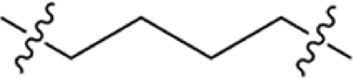	2.45 ± 0.11	8.07 ± 0.51
BST-5	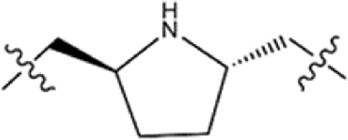	10.12 ± 1.15	17.83 ± 1.24
RVX-208	—	39.86 ± 3.72	no inhibition
CJ-1383	—	no inhibition	936 ± 14.87

### 3.3 Structure−activity relationships (SAR)

**FIGURE 5:**
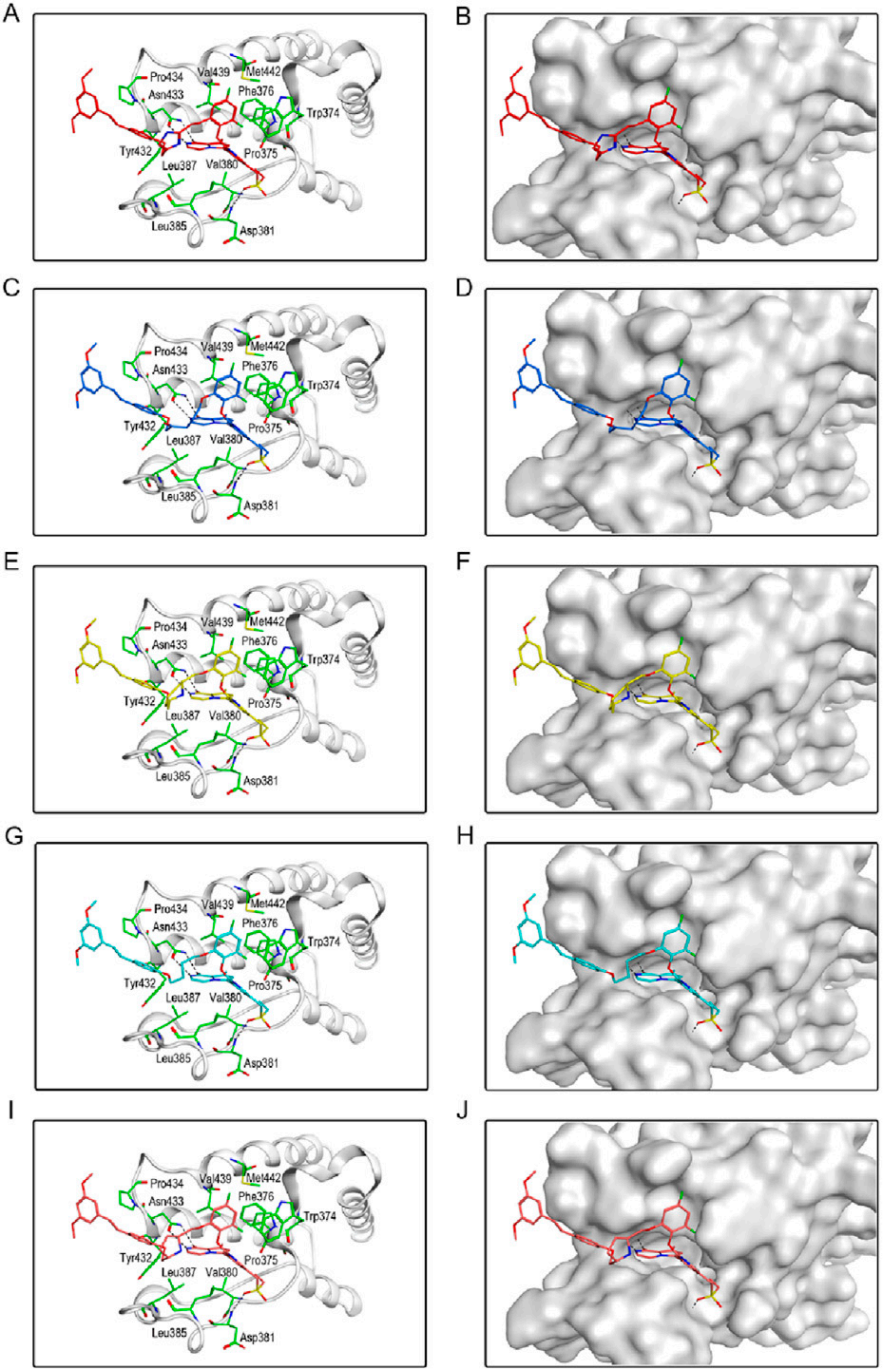
The binding mode of BSTs 1-5 in the active site of BRD4. **(A, B)** The binding mode of BST-1 (red sticks) in the active site of BRD4. **(C, D)** The binding mode of BST-2 (blue sticks) in the active site of BRD4. **(E, F)** The binding mode of BST-3 (yellow sticks) in the active site of BRD4. **(G, H)** The binding mode of BST-4 (cyan sticks) in the active site of BRD4. **(I, J)** The binding mode of BST-5 (orange sticks) in the active site of BRD4. Residues in the active site are shown as green sticks. BRD4 is colored in light grey. The hydrogen bonds are represented in black dashed lines.

We individually analyzed the docking patterns and interactions of BSTs 1-5 with BRD4 and STAT3. Figure 5 displayed the interactions between BSTs 1-5 and BRD4. These BSTs 1-5 contained an 8-methylpyrrolo [1,2-a] pyrazin-1(2H)-one structure, of which the oxygen and nitrogen atoms of the amide group formed a pair of hydrogen bonds with the nitrogen and oxygen atoms of the amide group on the side chain of the key residue Asn433 in BRD4. Additionally, These BSTs 1-5 contained a diethylsulfone group. The oxygen atom of this group formed a hydrogen bond with the nitrogen atom of the key residue Asp381 of BRD4. Therefore, the core structures of BSTs 1-5 formed hydrogen bonds with the key residues Asn433 and Asp381 at the binding site of BRD4, indicating that BSTs 1-5 could specifically bind to BRD4. Among them, BST-4 had a butyl group in its structure, which formed stronger hydrophobic interactions with the key residues Tyr432, Val380, Leu387, Pro434, Val439, and Leu385 at the BRD4 binding site. It suggested that among the five compounds, BST-4 exhibited the best inhibitory activity against BRD4.

**FIGURE 6:**
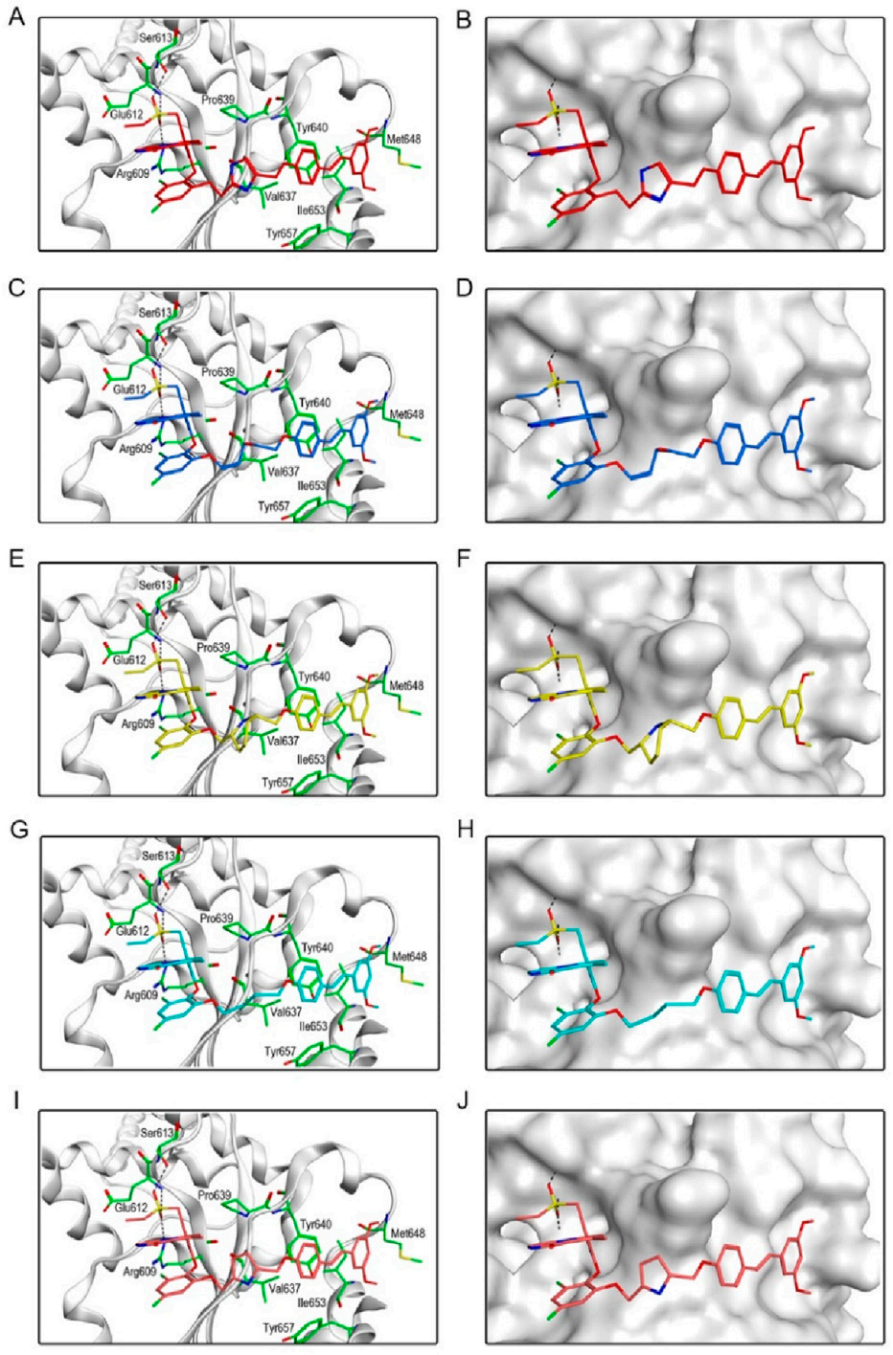
The binding mode of BSTs 1-5 in the active site of STAT3. **(A, B)** The binding mode of BST-1 (red sticks) in the active site of STAT3. **(C, D)** The binding mode of BST-2 (blue sticks) in the active site of STAT3. **(E, F)** The binding mode of BST-3 (yellow sticks) in the active site of STAT3. **(G, H)** The binding mode of BST-4 (cyan sticks) in the active site of STAT3. **(I, J)** The binding mode of BST-5 (orange sticks) in the active site of STAT3. Residues in the active site are shown as green sticks. STAT3 is colored in light grey. The hydrogen bonds are represented in black dashed lines.

Figure 6 showed the interactions between BSTs 1-5 and STAT3. The oxygen atom on the diethylsulfone group of BSTs 1-5 formed a hydrogen bond with the nitrogen atom of the guanidinium group on the side chain of Arg609 and with the nitrogen atom of the key residue Glu612. Moreover, another oxygen atom on the diethylsulfone group of BSTs 1-5 formed a hydrogen bond with the hydroxyl group of the side chain of Ser613. Thus, these BSTs 1-5 specifically bound to the active site of STAT3 and had an inhibitory effect on STAT3. Among them, the butyl group in the structure of BST-4 formed stronger hydrophobic interactions with the key residues Pro639, Tyr640, Val637, and Tyr657 at the STAT3 binding site. The results indicate that BST-4 had the best inhibitory activity against STAT3 among the five compounds.

In summary, these interaction modes demonstrate that BSTs 1-5 stably bound by interacting with key residues of BRD4 and STAT3. Specifically, the butyl group of BST-4 was able to form stronger hydrophobic interactions with key residues of both BRD4 and STAT3, resulting in BST-4 showing enhanced inhibitory activity against both BRD4 and STAT3.

### 3.4 MD simulations

**FIGURE 7:**
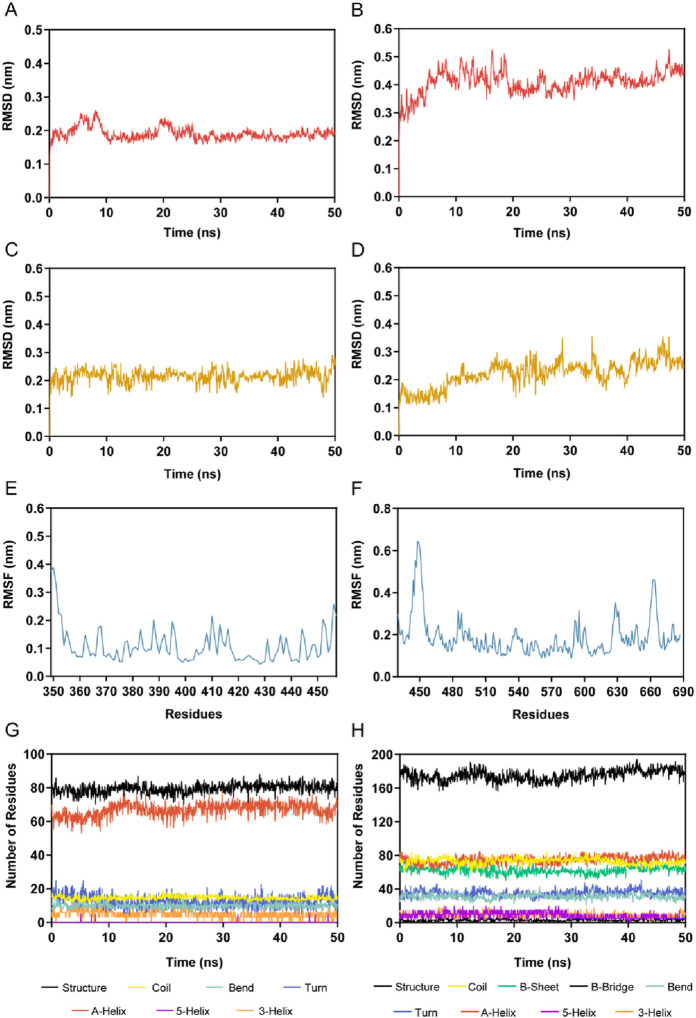
MD simulation of BST-4 in complex with BRD4 and STAT3. **(A)** The backbone RMSD of the complex of BRD4 and BST-4. **(B)** The backbone RMSD of the complex of STAT3 and BST-4. **(C)** RMSD of FH-5 atoms in the complex of BRD4 and BST-4. **(D)** RMSD of BST-4 atoms in the complex of STAT3 and BST-4. **(E)** RMSF of BRD4 Cα atoms in the complex of BRD4 and BST-4. **(F)** RMSF of STAT3 Cα atoms in the complex of STAT3 and BST-4. **(G, H)** The secondary structures analysis of BRD4 and STAT3, respectively.

We further explored the binding stability of BRD4-BST-4 and STAT3-BST-4 complexes by MD simulations. Figures 7A, B illustrated the RMSD plots (nm) of the BRD4-BST-4 and STAT3-BST-4 complexes for the atoms in the MD simulation running for 50 ns, respectively. After 25 ns, the RMSD of the BRD4-BST-4 reached stability at roughly 0.2 nm. The RMSD of STAT3-BST-4 had risen initially and eventually stabilized between about 0.3 nm and 0.5 nm. The results showed stable binding between the BST-4 and BRD4/STAT3. In addition, the results of the RMSD of BST-4 further demonstrated the dynamic stability of the ligand in the complexes (Figures 7C, D). Next, during the 50 ns MD simulations, the residual flexibility of BRD4 and STAT3 was observed by analyzing the root mean square fluctuation (RMSF) of all atoms. As depicted in Figures 7E, F, the key residues of BRD4 and STAT3 have small fluctuations throughout the simulation process. In addition, Figures 7G, H presented that the secondary structures of BRD4 and STAT3 hardly changed significantly during the simulation. Therefore, our docking and MD results indicated that BST-4 could interact with the critical site residues of BRD4 and STAT3 with notable binding stability.

### 3.5 Binding activity of BSTs 1-5 with BRD4 and STAT3

To elucidate the significant inhibition of RCC cells by BSTs 1-5 targeting BRD4 and STAT3 proteins, MST experiments were conducted to verify the direct binding of BSTs 1-5 to these proteins. The results are presented in Table 2; Supplementary Figure S1. BSTs 1-5 exhibited strong binding affinity at the nanomolar level. When compared to RVX-208 (*K*_d_ = 24.84 ± 2.31 nM) and CJ-1383 (*K*_d_ = 905 ± 12.67 nM), BSTs 1-5 showed enhanced binding affinity to BRD4 (*K*_d_ = 1.46–12.25 nM) and STAT3 proteins (*K*_d_ = 4.28–28.74 nM). Notably, BST-4 displayed the most potent binding, with affinities 17-fold and 211-fold greater than those of RVX-208 and CJ-1383, respectively. Subsequently, we tested the binding activity of BST-4 in HEK293T cells by NanoBRET assay. As shown in Supplementary Figure S3, BST-4 exhibited good inhibitory activity in cells against BRD4 (IC_50_ = 2.58 ± 0.07 nM) and STAT3 (IC_50_ = 5.17 ± 0.09 nM), respectively. This demonstrates that BST-4 binds to both BRD4 and STAT3 in cells.

**TABLE 2:** The binding affinities of BSTs 1-5, RVX-208 and CJ-1383 to BRD4 and STAT3.

Compounds	BRD4 (*K*_d_, nM)	STAT3 (*K*_d_, nM)
BST-1	12.25 ± 1.16	16.43 ± 0.95
BST-2	3.62 ± 0.25	28.74 ± 3.71
BST-3	5.37 ± 0.49	8.42 ± 0.38
BST-4	1.46 ± 0.07	4.28 ± 0.59
BST-5	9.72 ± 0.84	13.73 ± 0.98
RVX-208	24.84 ± 2.31	no inhibition
CJ-1383	no inhibition	905 ± 12.67

In addition, we tested the affinity of BST-4 for other proteins of the BET and STAT families. The results showed that BST-4 did not significantly interact with other proteins of the BET and STAT families (Supplementary Table S1). This result demonstrates the accuracy of our screening method. This high selectivity may stem from the precise construction of the BRD4-based pharmacophore model, which accurately captures the key interaction features of BRD4. Based on the above results, we further discuss the efficacy and selectivity of BST-4 with respect to the existing therapeutic agents.RVX-208 and OTX-015 are BRD4 inhibitors that are currently in clinical trials, of which OTX-015 showed IC_50_ value of approximately 92 nM as reported (Boi et al., 2015). BST-4 showed higher inhibitory activity compared to RVX-208 and OTX-015. In terms of selectivity, OTX-015 inhibited BRD2, BRD3, and BRD4, with no significant selectivity for BRD4. BST-4 showed excellent selectivity while having low nanomolar inhibition. In addition, in targeting STAT3, BST-4 showed significantly enhanced inhibition of STAT3 compared with CJ-1383. However, the selectivity of CJ-1383 treatment is currently unclear. BST-4 was selective for both BRD4 and STAT3 while exhibiting efficient inhibitory activity. Therefore, the high efficiency and specificity of BST-4 may be significant in drug development.

### 3.6 Kinase selectivity profile of BST-4

To further validate the selectivity of BST-4 on both BRD4 and STAT3, we performed a panel of 76 human kinases inhibitory assay on BST-4. As shown in Table 3, the inhibitory effect of BST-4 on these kinases was negligible (IC_50_ > 10 μM). BST-4 showed less than 10% inhibition of all 76 kinases at 1 µM (Supplementary Table S2). We then tested the selectivity of BST-4 for BET and STAT family members to demonstrate target specificity. The results showed that BST-4 did not significantly inhibit other BET and STAT family members (Table 4). Overall, these data suggest that BST-4 is a dual inhibitor of STAT3 and BRD4, with no selectivity for other targets.

**TABLE 3:** Selectivity testing of BST-4 on a panel of 76 tyrosine kinases.

Target	IC_50_ (μM)	Target	IC_50_ (μM)	Target	IC_50_ (μM)	Target	IC_50_ (μM)
ABL1	>10	EPHA8	>10	HCK	>10	NTRK3	>10
ABL2	>10	EPHB1	>10	IGF1R	>10	PDGFRA	>10
ALK	>10	EPHB2	>10	INSR	>10	PDGFRB	>10
AXL	>10	EPHB3	>10	INSRR	>10	PTK2	>10
BLK	>10	EPHB4	>10	ITK	>10	PTK2B	>10
BMX	>10	ERBB2	>10	JAK1	>10	PTK6	>10
BTK	>10	ERBB4	>10	JAK2	>10	RET	>10
CSF1R	>10	FER	>10	JAK3	>10	ROS1	>10
CSK	>10	FES	>10	KDR	>10	SRC	>10
DDR1	>10	FGFR1	>10	KIT	>10	SRMS	>10
DDR2	>10	FGFR2	>10	LCK	>10	SYK	>10
EGFR	>10	FGFR3	>10	LTK	>10	TEC	>10
EPHA1	>10	FGFR4	>10	LYN	>10	TEK	>10
EPHA2	>10	FGR	>10	MERTK	>10	TNK2	>10
EPHA3	>10	FLT1	>10	MET	>10	TXK	>10
EPHA4	>10	FLT3	>10	MST1R	>10	TYK2	>10
EPHA5	>10	FLT4	>10	MUSK	>10	TYRO3	>10
EPHA6	>10	FRK	>10	NTRK1	>10	YES1	>10
EPHA7	>10	FYN	>10	NTRK2	>10	ZAP70	>10

**TABLE 4:** The inhibitory effect of BST-4 on other members of the BET and STAT families.

Targets	IC_50_ (μM)	Targets	IC_50_ (μM)
BRD2	>10	STAT4	>10
BRD3	>10	STAT5a	>10
BRDT	>10	STAT5b	>10
STAT1	>10	STAT6	>10
STAT2	>10		

### 3.7 Evaluation of *in vitro* anti-proliferative activity

**FIGURE 8:**
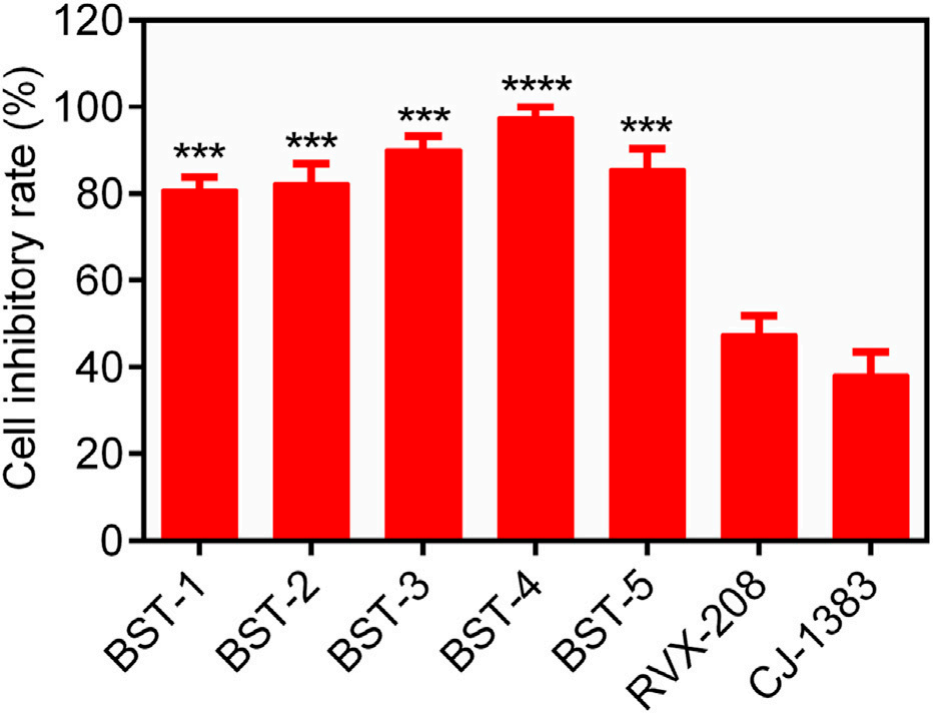
The cell inhibitory rate (%) of BSTs 1-5 against CAKI-2 cells. RVX-208 and CJ-1383 are used as a positive control. Data are presented as the mean ± SD, n = 3. The *P* value was determined by a 2-tailed paired t-test: ***p < 0.001 vs RVX-208 and ****p < 0.0001 vs RVX-208.

We further investigated the antiproliferative activity of BSTs 1-5 on CAKI-2 cells and measured their inhibition rate on the cells. As exhibited in Figure 8, BSTs 1-5 displayed stronger inhibitory effects on CAKI-2 cells compared to RVX-208 and CJ-1383, with cell inhibition rates above 80%. Importantly, BST-4 had the highest *in vitro* CAKI-2 cells inhibitory capacity, which was consistent with the results of the enzymatic inhibition assays described above. Furthermore, the values of IC_50_ of BST-4 against more RCC cell lines were determined. The IC_50_ values of BST-4 against cancer cell lines were listed in Table 5. BST-4 exhibited effective cell growth inhibition on cancer cells, except for normal renal cortical proximal tubular epithelial cell HK-2 and PBMCs cells (IC_50_ > 10 μM). In particular, BST-4 showed the best inhibitory effect on CAKI-2 cells with an IC_50_ value of 0.76 ± 0.05 μM (Supplementary Figure S2). Overall, these results indicate that BST-4 exerted highly potent inhibitory activity *in vitro* against multiple types of renal cancer cells. Importantly, BST-4 has selective anti-tumor activity. BST-4 effectively inhibited the growth of tumor cells at lower concentrations with less effect on normal cells, which helps to reduce systemic toxicity during treatment. This is crucial for reducing the side effects of drugs and improving the safety of treatment, which lays a good foundation for its clinical application.

**TABLE 5:** Cytotoxicity of BST-4 against RCC cell lines and normal cell line.

Name	IC_50_ (μM) ± SD^a^						
	CAKI-2	RCC4	SLR-23	769-P	A704	HK-2	PBMCs
BST-4	0.76 ± 0.05	2.19 ± 0.64	1.52 ± 0.43	1.68 ± 0.32	1.07 ± 0.18	>10	>10

^a^ IC_50_ (μM) is the concentration of compound needed to reduce cell growth by 50% following 48 h cell treatment with BST-4. Each experiment was performed at least three times. Data are presented as the mean ± SD.

To validate that dual inhibition of BRD4 and STAT3 has synergistic effect in inhibition cancer cell growth, we tested the combined inhibition of BRD4 (RVX-208) and STAT3 (CJ-1383) inhibitors. The combined inhibition of CAKI-2 cells by both BRD4 (RVX-208) and STAT3 (CJ-1383) inhibitors (IC_50_ = 0.93 ± 0.08 μM) was significantly more effective than that of the individual BRD4 (RVX-208) inhibitors (IC_50_ = 3.71 ± 0.42 μM) or STAT3 (CJ-1383) inhibitors (IC_50_ = 6.86 ± 0.54 μM) (Supplementary Table S3). The results support the rational to develop small molecules that dual-targeting BRD4/STAT3. In addition, the cell growth inhibitory effect of BST-4 was more effective than that of the combination of RVX-208 and CJ-1383.

Furthermore, to demonstrate whether the growth inhibition effect of BST-4 was dependent on BRD4 and STAT3, non-targeting shRNA (shControl), BRD4-targeting shRNA (shBRD4), STAT3-targeting shRNA (shSTAT3), and BRD4 and STAT3-targeting shRNA (shBRD4/STAT3)-transfected CAKI-2 cells were constructed, and then MTT assay was used to detect the cytotoxicity of BST-4 to these cells. As shown in Supplementary Table S4, BST-4 exhibited stronger cytotoxicity to shControl-CAKI-2 cells (IC_50_ = 0.76 ± 0.05 μM) than shBRD4-CAKI-2 (IC_50_ = 5.09 ± 0.27 μM), shSTAT3-CAKI-2 (IC_50_ = 3.48 ± 0.21 μM), and shBRD4/STAT3-CAKI-2 (IC_50_ > 10 μM) cells, indicating that the growth inhibition activity of BST-4 is dependent on BRD4 and STAT3.

### 3.8 Effects on c-myc expression and STAT3 phosphorylation

**FIGURE 9:**
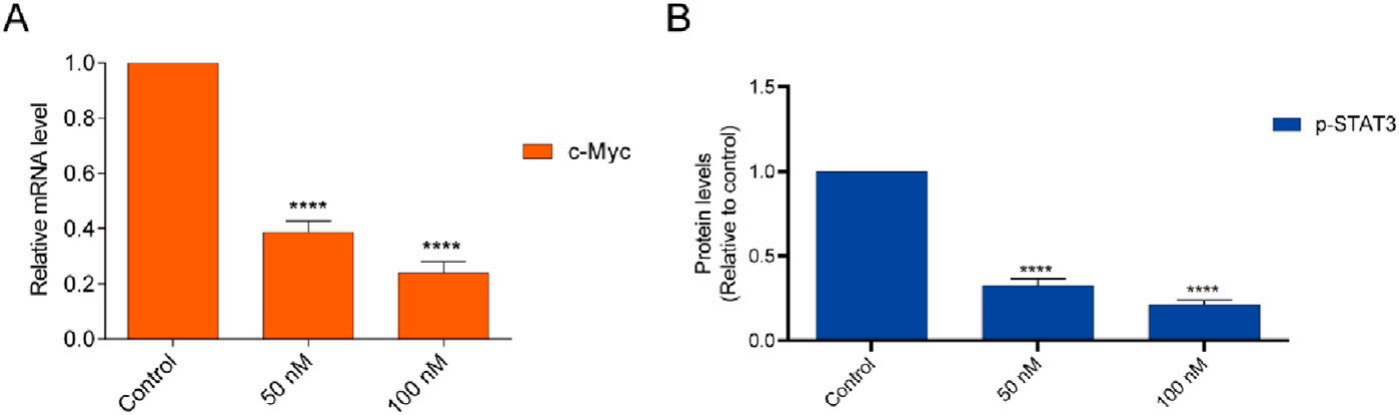
**(A)** Inhibition of the BST-4 on the expression levels of c-myc mRNA on CAKI-2 cells by RT-qPCR analyses. **(B)** BST-4 inhibited STAT3 phosphorylation levels on CAKI-2 cells via Western blot assay. GADPH was used as a loading control. The data were expressed as mean ± SD, n = 3, ****p < 0.0001.

The BRD4 inhibitor induces an antiproliferative effect associated with the downregulation of c-myc transcription. Quantitative real-time PCR (RTqPCR) was performed to study the cellular effect of BST-4 related to c-myc. As shown in Figure 9A, BST-4 strongly dose-dependent downregulated the expression of c-myc mRNA. To detect whether BST-4 displayed antitumor activity against osteosarcoma cells by suppressing the phosphorylation of STAT3, CAKI-2 were treated with BST-4, for 24h and Western blotting analysis was performed to examine the expression of phosphorylated STAT3 *in vitro*. As presented in Figure 9B, BST-4 significantly decreased STAT3 phosphorylation at a concentration of 100 nM. These data suggest that the antiproliferation effects of BST-4 may through the inhibition of the STAT3 phosphorylation and BRD4-dependent pathway. Based on these results, we further discussed the mechanism of action of the BST-4-induced cell signaling pathway. Intracellular c-myc protein levels are directly regulated by BRD4. BST-4 inhibits BRD4-mediated expression of the key oncogene c-myc by inhibiting the bromodomain of BRD4 and blocking its binding to acetylated histones. It disrupts the high expression of c-myc *in vivo* and suppresses c-myc transcription in tumor cells. BST-4 inhibits STAT3 leading to reduced levels of STAT3 phosphorylation. Thus, BST-may block the JAK-STAT3 signaling pathway, thereby inhibiting STAT3-mediated gene expression. In summary, BST-4, as a dual-target inhibitor, affects c-myc expression and STAT3 phosphorylation by inhibiting the activities of BRD4 and STAT3. However, the full impact of BST-4 on cell signaling pathways still requires further in-depth studies.

### 3.9 *In vivo* antitumor effects

**FIGURE 10:**
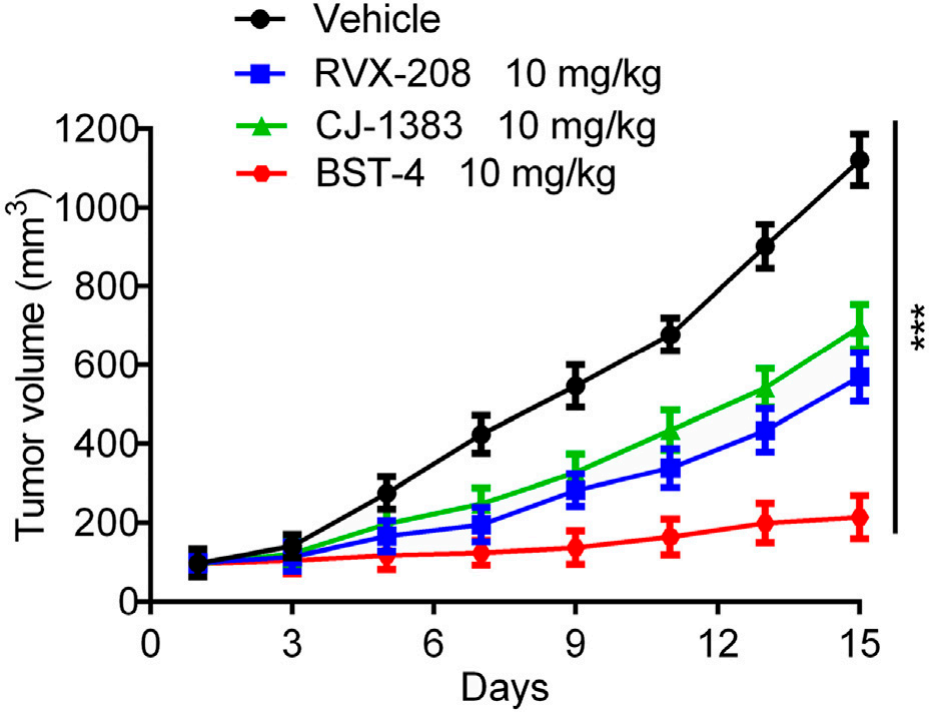
*In vivo* antitumor effect of BST-4. Nude mice bearing tumor were randomly divided into four groups, and treated with RVX-208, CJ-1383 and BST-4, all at a dosage of 10 mg/kg or vehicle. Tumor volumes were measured and calculated once every 2 days. Data are presented as the mean ± SD, n = 5. ***p < 0.001 means a significant difference versus the vehicle group.

**FIGURE 11:**
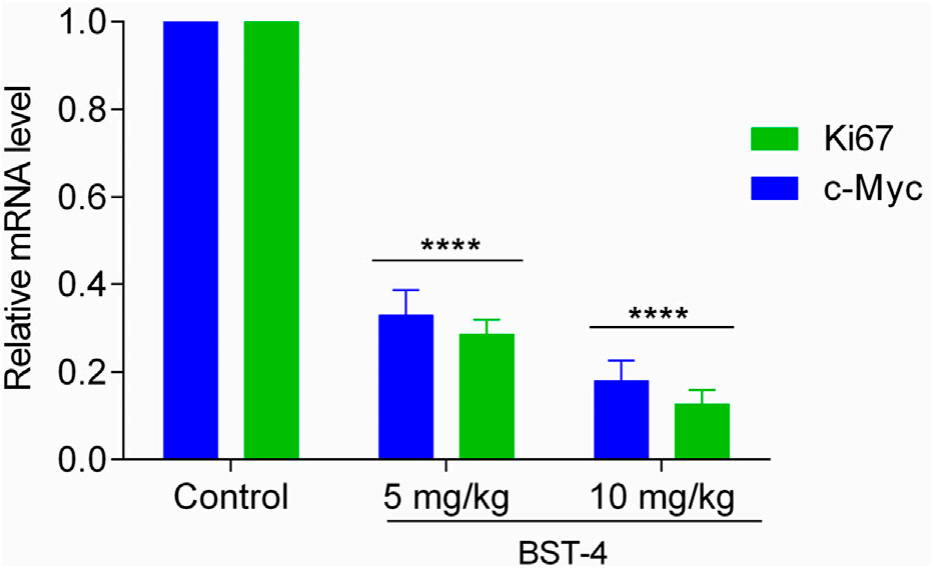
Inhibition of the BST-4 on the expression levels of c-myc mRNA and Ki67 on the tumor tissues by RT-qPCR analyses. The data were expressed as mean ± SD, n = 3, ****p < 0.0001.

**FIGURE 12:**
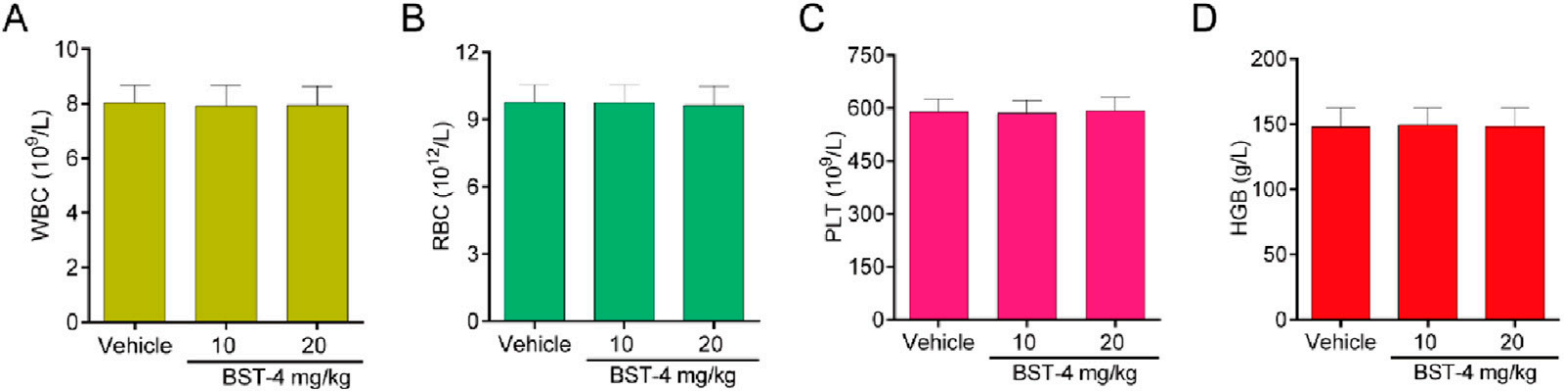
Hematology parameters of mice in the BST-4-treated groups and vehicle groups. **(A)** White blood cell (WBC); **(B)** Red blood cells (RBC); **(C)** Platelets (PLT); **(D)** Hemoglobin (HGB). Data are presented as the mean ± SD, n = 5.

A CAKI-2 cell-derived xenograft model was established in order to further assess the *in vivo* activity of BST-4. Mice were separated into vehicle group and drug-treated groups: RVX-208 (10 mg/kg), CJ-1383 (10 mg/kg), and BST-4 (10 mg/kg). Treatment with BST-4 significantly inhibited tumor growth in mice (Figure 10). Moreover, we observed that the BST-4-treated group had a more remarkable inhibitory effect on tumor volume growth in mice in comparison to the RVX-208 and CJ-1383 treated groups. In summary, the results of these experiments suggested that BST-4 suppressed the growth of tumor, and could be a promising lead compound for RCC therapy. We next tested whether the molecular pathways we discovered *in vitro* were relevant *in vivo*. We examined the effect of BST4 on the *in vivo* mRNA levels of c-myc and Ki67. As shown in Figure 11, BST4 downregulated the expression levels of c-myc mRNA and Ki67 mRNA *in vivo*. Similarly, the Western blot analysis results indicated that the protein expression levels of p-STAT3 and c-myc were significantly downregulated after BST-4 treatment (Supplementary Figure S4). Moreover, the levels of representative hematological markers also showed no significant differences in the BST-4-treated groups and vehicle groups (Figure 12). These data suggest that BST-4 has no obvious adverse toxic effects. In conclusion, BST-4, as a novel dual-target BRD4/STAT3 inhibitor, exhibited significant antitumor activity and safety. Therefore, BST-4 has important potential for clinical application. In the future, it is expected that further preclinical studies and clinical trials will be conducted to evaluate the safety and efficacy of BST-4 in therapy and provide new options for cancer treatment.

## 4 Conclusion

At present, the diagnostic rate of RCC is rapidly increasing every year. Dual-targeting drugs provide a new strategy for developing novel anticancer drugs. Here, we discovered novel dual-targeted BRD4/STAT3 inhibitors through a combined screening protocol. In particular, BST-4 had the highest inhibitory activity toward both BRD4 (IC_50_ = 2.45 ± 0.11 nM) and STAT3 (IC_50_ = 8.07 ± 0.51 nM). In addition, MD simulation confirmed the dynamic structural stability of BST-4 combined with BRD4 and STAT3. The cell proliferation inhibition experiments confirmed that BST-4 had significant antiproliferative activity on certain RCC cell lines (CAKI-2, RCC4, SLR-23, 769-P, and A704), especially CAKI-2, while it had a slight inhibitory activity on normal human kidney cells HK-2, indicating that it had few significant toxic side effects. In particular, BST-4 has an inhibition rate of more than 95% against CAKI-2 cells. Meanwhile, *in vivo* experiments showed that BST-4 exhibits more effective antitumor activity in CAKI-2 xenografts than BRD4 inhibitor RVX-208 and STAT3 inhibitor CJ-1383. Notably, we found that the docking score was consistent with the cytotoxicity test result. In our virtual screening prediction, BST-4 with the lowest docking score demonstrated the greatest biological validation, which confirmed the rationality of calculating docking predictions. In summary, we have successfully discovered a potent and safe antitumor drug targeting BRD4/STAT3 through a structure based virtual screening scheme, and was verified through both *in vitro* and *in vivo* biological experiments.

## Data Availability

The original contributions presented in the study are included in the article/Supplementary Material, further inquiries can be directed to the corresponding authors.
