# Supplementary material for: A combinatorial screening protocol for identifying novel and highly potent dual-target inhibitor of BRD4 and STAT3 for kidney cancer therapy
Source: Front Pharmacol. 2025 Feb 26;16:1560559. doi: 10.3389/fphar.2025.1560559 (PMC11897524; doi:10.3389/fphar.2025.1560559)
Supplement: Supplementary file 1 [file DataSheet1.docx]

**Supplementary Material**

**A combinatorial screening protocol for identifying novel and highly potent dual-target inhibitor of BRD4 and STAT3 for kidney cancer therapy**

**
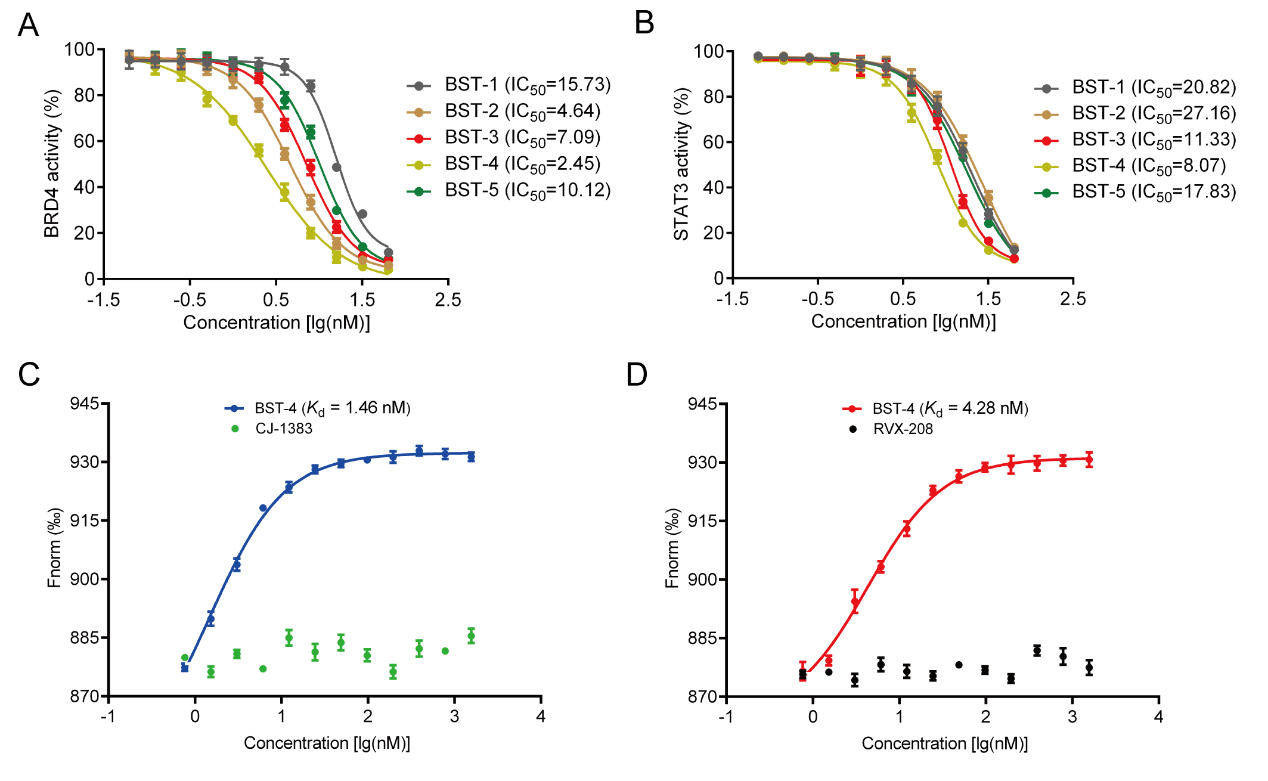
**

**Figure S1.** Concentration-response curves for IC_50_ values of BST 1-4 inhibition of BRD4 (A) and STAT3 (B). Concentration-response curves of *K*_d_ values of BST-4 to BRD4 (C) and STAT3 (D) by MST assay. Data are presented as the mean ± SD (n = 3).

**

**

**Figure S2.** Cell proliferation inhibition curve of CAKI-2 cells treated with BST-4





**Figure S3.** Inhibition of the BST-4 on the expression of c-myc and p-STAT3 levels on the tumor tissues via Western blot assay. GADPH was used as a loading control. The data were expressed as mean ± SD, n = 3, **** p < 0.0001.

**Table S1.** The binding affinities of BST-4 on other members of the BET and STAT families.

| **Targets** | ***K*_d_ (μM)** | **Targets** | ***K*_d_ (μM)** |
| --- | --- | --- | --- |
| BRD2 | > 10 | STAT4 | > 10 |
| BRD3 | > 10 | STAT5a | > 10 |
| BRDT | > 10 | STAT5b | > 10 |
| STAT1 | > 10 | STAT6 | > 10 |
| STAT2 | > 10 |  |  |

**Table S2.** Kinase selectivity profile of BST-4 at a concentration of 1 µM on 76 tyrosine kinases.

| **Target** | **%inhibition at 1 μM** | **Target** | **%inhibition at 1 μM** | **Target** | **%inhibition at 1 μM** | **Target** | **%inhibition at 1 μM** |
| --- | --- | --- | --- | --- | --- | --- | --- |
| ABL1 | 0.31 | EPHA8 | 0.72 | HCK | 0.86 | NTRK3 | 4.75 |
| ABL2 | 0.44 | EPHB1 | 3.35 | IGF1R | 0.91 | PDGFRA | 0.37 |
| ALK | 0.72 | EPHB2 | 0.51 | INSR | 2.53 | PDGFRB | 0.10 |
| AXL | 1.83 | EPHB3 | 0.98 | INSRR | 0.62 | PTK2 | 1.48 |
| BLK | 0.54 | EPHB4 | 0.97 | ITK | 0.75 | PTK2B | 0.73 |
| BMX | 3.05 | ERBB2 | 4.43 | JAK1 | 1.21 | PTK6 | 2.34 |
| BTK | 0.11 | ERBB4 | 0.78 | JAK2 | 0.18 | RET | 0.41 |
| CSF1R | 2.02 | FER | 0.03 | JAK3 | 0.39 | ROS1 | 0.97 |
| CSK | 0.74 | FES | 0.05 | KDR | 1.51 | SRC | 3.24 |
| DDR1 | 3.63 | FGFR1 | 0.59 | KIT | 0.25 | SRMS | 0.53 |
| DDR2 | 0.70 | FGFR2 | 0.45 | LCK | 0.77 | SYK | 1.92 |
| EGFR | 0.37 | FGFR3 | 2.84 | LTK | 0.21 | TEC | 0.67 |
| EPHA1 | 0.02 | FGFR4 | 0.68 | LYN | 1.96 | TEK | 4.16 |
| EPHA2 | 0.50 | FGR | 0.28 | MERTK | 0.07 | TNK2 | 0.93 |
| EPHA3 | 0.89 | FLT1 | 0.14 | MET | 0.68 | TXK | 0.09 |
| EPHA4 | 0.58 | FLT3 | 0.52 | MST1R | 5.02 | TYK2 | 5.33 |
| EPHA5 | 3.17 | FLT4 | 1.82 | MUSK | 1.26 | TYRO3 | 0.04 |
| EPHA6 | 0.26 | FRK | 0.95 | NTRK1 | 0.67 | YES1 | 1.22 |
| EPHA7 | 0.91 | FYN | 2.03 | NTRK2 | 1.22 | ZAP70 | 0.68 |

**Table S3.** The growth inhibitory activity of BST-4 and individual BRD4 (RVX-208) or STAT3 (CJ-1383) inhibitors, or a combination of both on CAKI-2 cells.

| Cancer cells | IC_50_ (μM) ± SD*^a^* | | | | |
| --- | --- | --- | --- | --- | --- |
|  | RVX-208 | CJ-1383 | BST-4 | RVX-208+CJ-1383 |  |
| CAKI-2 | 3.71 ± 0.42 | 6.86 ± 0.54 | 0.76 ± 0.05 | 0.93 ± 0.08 |  |

*^a^*IC_50_ (μM) is the concentration of compound needed to reduce cell growth by 50% following 48 h cell treatment with compound BST-4. Each experiment is performed at least three times. Data are presented as the mean ± SD.

**Table S4.** The sensitivity of BST-4 to CAKI-2 cells stably expressing shRNA detected by MTT assay.

| Name | IC_50_ (μM) ± SD*^a^* | | | |
| --- | --- | --- | --- | --- |
|  | shControl-CAKI-2 | shBRD4-CAKI-2 | shSTAT3-CAKI-2 | shBRD4/STAT3-CAKI-2 |
| BST-4 | 0.76 ± 0.05 | 5.09 ± 0.27 | 3.48 ± 0.21 | > 10 |

*^a^*IC_50_ (μM) is the concentration of compound needed to reduce cell growth by 50% following 48 h cell treatment with BST-4. shControl-CAKI-2: CAKI-2 cells stably expressing Control shRNA; shBRD4-CAKI-2 cells: CAKI-2 cells stably expressing BRD4 shRNA; shSTAT3-CAKI-2 cells: CAKI-2 cells stably expressing STAT3 shRNA; shBRD4/STAT3-CAKI-2 cells: CAKI-2 cells stably expressing BRD4/STAT3 shRNA. Each experiment was performed at least three times. Data are presented as the mean ± SD.
